# Direct Use of the Savitzky–Golay Filter to Develop an Output-Only Trend Line-Based Damage Detection Method

**DOI:** 10.3390/s20071983

**Published:** 2020-04-02

**Authors:** Hadi Kordestani, Chunwei Zhang

**Affiliations:** Structural Vibration Group, Qingdao University of Technology, Qingdao 266033, China; hadi@qut.edu.cn

**Keywords:** Savitzky–Golay filter, trend line, damage detection, moving sprung mass, baseline free

## Abstract

The Savitzky–Golay filter (SGF) is a time-domain technique that determines a trend line for a signal. The direct application of SGF for damage localization and quantification is investigated in this paper. Therefore, a single-stage trend line-based damage detection method employing SGF is proposed in which the damage is located and quantified at the bridge under moving load. A simply supported beam under moving sprung mass is numerically simulated to verify the proposed method. Four different velocities and five different single- and multi-damage scenarios are considered. The acceleration data along the beam are obtained, manually polluted with noise and their trend lines are then determined using SGF. The results show that the proposed method can accurately locate and quantify the damage using these trend lines. It is proved that the proposed method is insensitive to the noise and velocity variation in which having a constant velocity is a hard task before and after damage. Additionally, defining a normalization factor and fitting a Gaussian curve to this factor provide an estimation for the baseline and therefore, it categorizes the proposed method as baseline-free method.

## 1. Introduction

Health monitoring of bridge type structures subjected to a moving load is a hot topic that has received much attention in the last few decades [1,2,3,4]. Although bridge visual inspection is a widely used approach due to its simplicity, it suffers from various disadvantages such as subjective judgement and difficult-to-access location issues [5]. Hence, systematic vibration-based bridge health monitoring (BHM) using a set of implemented sensors is a good alternative to overcome these limitations. Vibration-based BHM may rely on measuring both input excitations and output responses or only output responses. Since the recording of input excitations in a bridge is a difficult task due to practical issues, the output-only BHM is preferred. Transformers [6,7,8,9,10,11], time-domain methods [1,2,12,13,14,15,16,17], and source separation methods [18,19,20,21,22] are some of the vibration-based BHM approaches which were extensively addressed in the literature.

The effectiveness of transformers, such as the wavelet transformer, to locate the damage from bridge vibration data (e.g., stress or acceleration signals) was discussed by many researchers [6,7,8,9]. Hester and Gonzalez highlighted that wavelet analysis in a noisy environment can lead to inaccurate results [10]. Additionally, Zhu and Law illustrated that the road irregularities, velocity limitations, and wheel dimension have effects on the wavelet analysis results [11].

One of the famous example of time-domain techniques is random decrement technique (RDT), which aims to average the transient response of the signal out [2]. Lee et al. [14] calculated the free response of a bridge subjected to the traffic load using RDT. Kordestani et al. experimentally proved that RDT with a suitable energy-based damage index could be used for BHM systems [2]. A combination of RDT with empirical mode decomposition or source separation technique were also addressed [15,16]. Buff et al. [17] provided an educational systematic example to show how RDT can be employed for the health monitoring of a bridge.

Source separation approach is an output-only technique that determines the input excitations from output responses. It employs different techniques such as second order blind identification (SOBI) in which the modal parameters of structure are the side results of this approach. Loh et al. [21] used SOBI and successfully located the damage in a bridge structure. In the source separation methods, the number of modal parameters is limited to the number of sensors. A combination of SOBI with wavelet analysis was also reported to increase the determined modal parameters using a fixed number of sensors [22].

Based on the authors’ knowledge, there is a lack of investigation on the direct use of time-domain filters in the field of structural health monitoring (SHM). Moving average filter (MAF) is one of the famous time-domain filters that was frequently highlighted as a noise reduction filter [5,23,24]. Direct use of MAF was also numerically and experimentally addressed in which the damage was located along a simply supported beam under moving load [1,25]. Considering that MAF has zero-order polynomial function in its kernel, it can be categorized as Savitzky–Golay filter (SGF) family [26,27]. The SGF and its family mainly attempt to find a trend line for a signal. The application of SGF on enhancing/de-noising the results of damage detection techniques to detect the damage in the plate-like structure were experimentally illustrated as well [28,29,30].

The above literature mostly uses modal parameters such as the damage index (DI) which only locates the damage in bridge type structure. Moreover, their backgrounds are complex that make them time-consuming and costly. The authors previously proposed a double-staged BHM method using a combination of RDT and SGF [31]. Since the direct application of SGF on the BHM is relatively new topic that needs more investigation, this paper develops a single-stage output-only trend line-based damage detection/quantification method employing SGF in which an energy-based DI is used. This paper shows that a trend lines determined using SGF has only the first natural frequency of the bridge and can be used for damage localization purpose. Using an energy-based DI, the proposed method enables locating/quantifying the damage from these trend lines. A numerical model of simply supported beam under moving sprung mass with different velocities is used to verify the proposed method. The results show that the proposed method can accurately locate and quantify either the single damage or multiple damage. It should be noted that the proposed method is baseline free, very quick, simple to understand, and easy to use.

## 2. Basic Theory of SGF

This section describes the procedure of determining a trend line for a signal. Employing the least-square fit and a polynomial function as a filter kernel, SGF is able to reduce noises and find a trend line for a signal [26,27,32,33]. Suppose there are 2S+1 consecutive observations as $y_{i-S}$,$y_{i-S+1}$,*…*,$y_{i}$,*…*,$y_{i+S}$ from a signal $y_{t}$. The 2S+1 calls the SGF span which is discussed in Section 5.1. The filter kernel of SGF is a polynomial function as expressed below:

(1) $$F\left(\tau \right)=\sum_{j=0}^{j=r}\beta_{j}\tau^{j}=\beta_{0}+\beta_{1}\tau +\ldots +\beta_{r}\tau^{r}$$

where *r* and β are the order and coefficient of the polynomial function, respectively. There is no particular recommendation for selection of order and span of SGF. To use the least-square fit, the order of the polynomial function must be pre-determined, which is 3 in this paper. For the span of SGF, the authors recommend to choose it based on the first natural frequency, as follows: SGF span = (sampling frequency)/ (first natural frequency). The least-square fit calculates the coefficients of the polynomial function by minimizing the following expression:

(2) $$Min\left\{\sum_{\tau =-S}^{\tau =S}{\left(y_{i+\tau }-F\left(\tau \right)\right)}^{2}\right\}$$

SGF uses all the 2S+1 observations to calculate a fit and substitutes the value of $y_{i}$ with its fit at the point t=i. Therefore, for each data of the signal, $y_{i}$, a new fit has to be calculated. It should be mentioned that the MATLAB software provides a function to directly apply SGF to a signal.

## 3. Proposed Method

**Figure 1:**
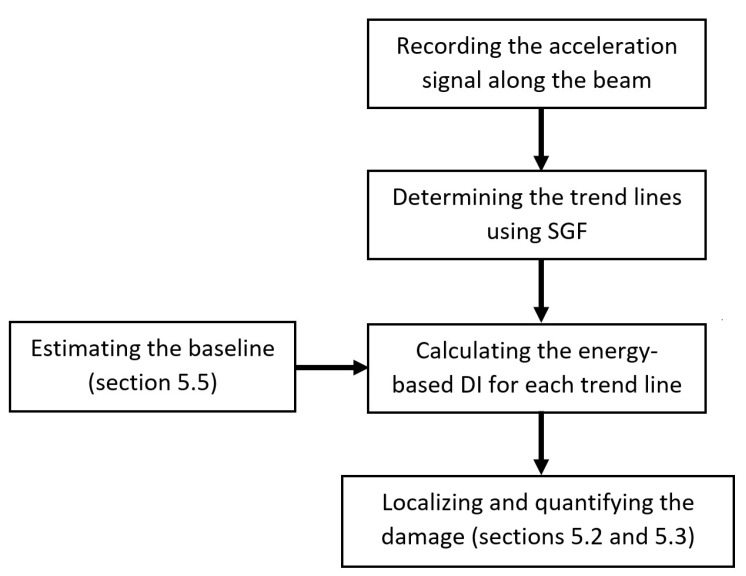
General flowchart of the proposed damage detection method.

Employing the SGF, an output-only baseline-free time-domain bridge damage detection method is developed in which the bridge acceleration response is used. Figure 1 schematically draws an overall view of the proposed method.

As shown in Figure 1, first the bridge acceleration response are recorded along the bridge subjected to the moving vehicle. Using SGF, a trend line for each individual bridge acceleration signal is then determined. The energy-based DI is calculated for each trend line in the next step. In this stage, the energy-based DI for non-damage condition can be estimated using the way that illustrated in Section 5.5. The location and severity of the possible damage can be determined as explained in Section 5.2 and Section 5.3.

## 4. The Numerical Model of Simply Supported Beam under Moving Sprung Mass

**Figure 2:**
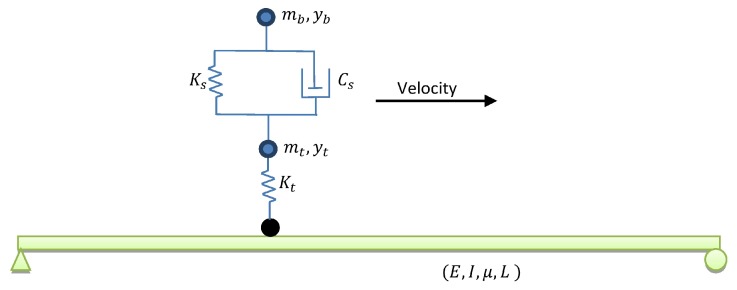
Schematic view of the simply supported beam under moving sprung mass.

The numerical investigation of simply supported beam under truck load was addressed by many researchers [1,5,25,31]. A truckload can numerically be modelled as a moving force, moving mass, or moving sprung mass. However, it is proved that the vibration response of a bridge under moving force is similar to the force vibration [34]. Moreover, the vehicle-bridge interaction shifts in the bridge natural frequencies [35,36,37]. Therefore, using moving sprung mass numerically represents a better model for vehicle-bridge interaction. An Euler-Bernoulli simply supported beam under a quarter car with different velocities was numerically modelled in this paper [25,31,38,39]. Figure 2 shows a schematic view of bridge under moving sprung mass. Table 1 and Table 2 provide the simply supported beam and quarter car details. For simplicity, the rotational degree of freedom of the car was restricted, so it can only vertically excite the bridge.

Finite Element Model (FEM) of a simply supported bridge under moving sprung mass was established in which the bridge and vehicle were considered with the detail shown in Table 1 and Table 2. To establish this FEM, ABAQUS software was used. In the first step, the gravity applied to the model, and the car then starts to move along the bridge at a constant velocity in the second step. The static and dynamic analysis were performed using full Newton algorithm. The vehicle was modelled using 3 reference points and two springs in which it can moves along the bridge. These three reference points refer to the body, axle and the contact between the wheel and the bridge. Therefore, the masses related to the body and axle were assigned to two of these reference points (in Figure 2, $m_{b}$ and $m_{t}$ are masses of body and axle). The interaction type in ABAQUS software was defined as frictionless-hard contact and it was assigned to the third reference point (the black circle in Figure 2). Therefore, the vehicle can easily vibrate in the vertical direction and move along the bridge. Typically, bridges are the low damped system [1,7,25] and therefore, the present paper ignores the damping in the bridge. Only vehicle has damper in its spring as described in Figure 2 and Table 2.

**Figure 3:**
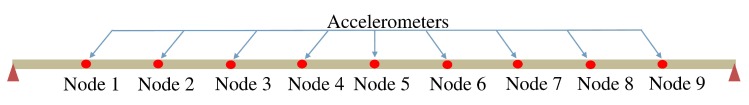
Schematic location of nodes to obtain acceleration data.

As listed in Table 2, the moving sprung mass passed the bridge at four different velocities. During each passage, the acceleration data were recorded at nine nodes along the bridge at the sampling frequency of 2000 Hz. Figure 3 shows these uniformly distributed nodes along the bridge. Five different damage scenarios were also considered, and are listed in Table 3. A rectangular profile was considered for the bridge section area. Decreasing the height of this profile can model the damage with different height ratio. The bridge length was divided into 500 finite elements and the crack was introduced to the bridge by decreasing the section area of two of these elements. The crack depth to the beam height ratio was considered to be the damage ratio.

## 5. Trend Lines and Damage Localization

### 5.1. Applying SGF on Each Acceleration Data

**Figure 4:**
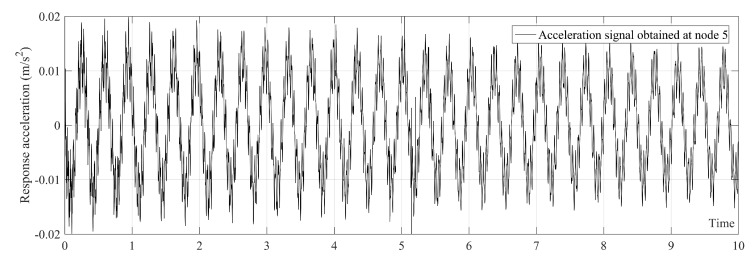
Acceleration response at the middle of the bridge under moving sprung mass with speed of 2.5 m/s.

An example of acceleration data obtained from node 5 (middle of the bridge) is shown in Figure 4. First the noise-free acceleration data are used to illustrate the proposed method. This could help the readers to understand the proposed method better. The noisy acceleration data are then used to prove that the proposed method is insensitive to the noise.

**Figure 5:**
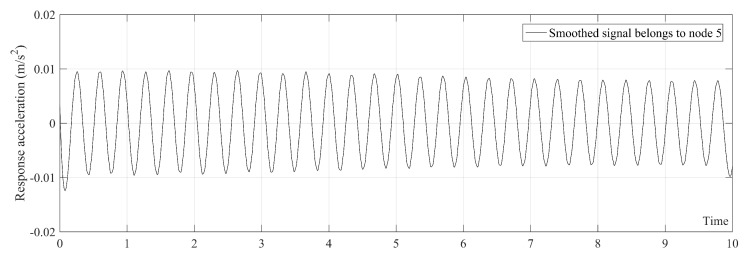
Trend line belongs to Figure 4.

As mentioned in Section 2, the order of the polynomial function used in the SGF was considered to be r = 3 in this paper. The authors found that selecting SGF span according to the first natural frequency of bridge (i.e., SGF span = sampling frequency/first natural frequency) leads to a trend line which shows the first natural frequency of the bridge. The first natural frequency of the simply supported beam in non-damage condition without the present of moving sprung mass is 2.9332 Hz. Therefore, SGF span = 2000/2.9332=681.85. Since the SGF span, 2S+1, should be considered to be an odd number, the SGF span in the case of non-damage condition is considered to be 683. The SGF span must be determined for each scenarios separately. The trend line of Figure 4 is plotted in Figure 5. Figure 5 clearly shows that the trend line only has the first natural frequency of the bridge which is 2.9332 Hz. The SGF is separately applied on all acceleration signals obtained from the bridge.

### 5.2. Damage Localization

**Figure 6:**
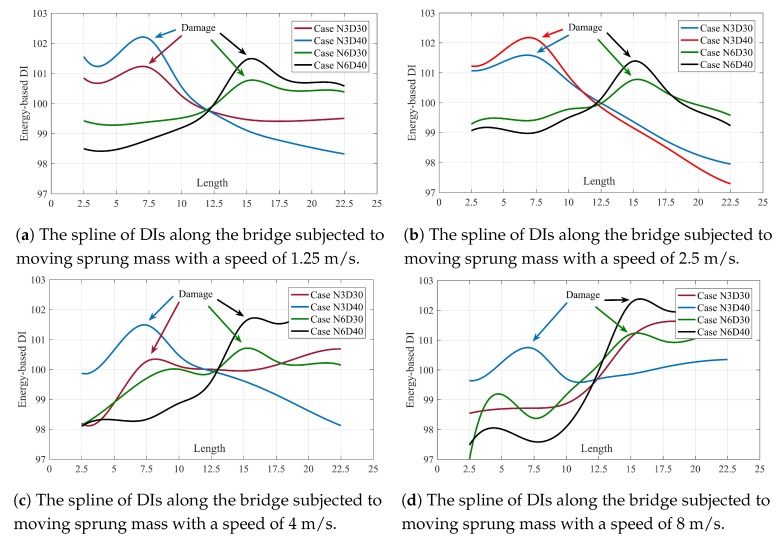
The spline of DIs along the bridge of different scenarios and different velocities. (**a**) velocity = 1.25 m/s, (**b**) velocity = 2.5 m/s, (**c**) velocity = 4 m/s, (**d**) velocity = 8 m/s.

**Figure 7:**
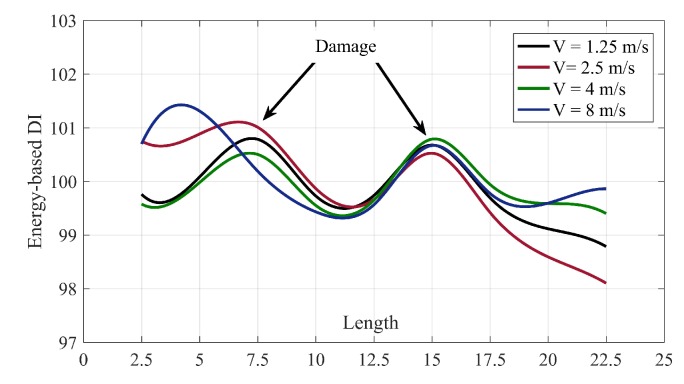
The spline of DIs along the bridge for the case of multi-damage, N3N6D40, with different velocities.

The insensitivity of DIs based on the modal parameters such as natural frequency were addressed by many researchers [5,40,41]. Choosing a proper DI from non-modal parameters can be either more sensitive or save time (because it does not need to solve the equation of motion) [2]. In this paper, in order to damage localization/quantification, an energy-based DI is employed [31,42]. To this end, the energy of signal can be determined using:

(3) $$E=\int {\left(Tr\right)}^{2}dt$$

where Tr is the trend line calculated using SGF. *E* is the energy of the signal. Obviously, the amplitude of the trend lines determined from different nodes of the bridge are different and need to be normalized as follow:

(4) $$\gamma_{i}=\left(\frac{E_{{IN}_{i}}}{\bar{E_{IN}}}\right)$$

where $E_{{IN}_{i}}$ is the energy of the trend line in non-damage condition at node *i* and $\bar{E_{IN}}$ is arithmetic mean of $E_{{IN}_{i}}$ calculated from all nodes. $\gamma_{i}$ is normalization factor. Therefore, an energy-based DI can be defined as:

(5) $${DI}_{i}=\left(\frac{E_{i}}{\bar{E}}\right)\times \frac{100}{\gamma_{i}}$$

where $E_{i}$ is the energy of the trend line at node *i* and $\bar{E}$ is arithmetic mean of $E_{i}$ calculated at all nodes. If there is no damage in the bridge, the DIs along the bridge keep constant value of 100. Damage changes the distribution of energy in the bridge, so it is supposed to have a pick at the energy of trend line at the vicinity of the damage. Figure 6 shows the DIs calculated along the bridge for different single damage scenarios. To have a better understanding, the DIs of each case in Figure 6 are connected to each other using splines. Figure 7 shows the multi-damage localization using proposed DI.

Figure 6 and Figure 7 prove that the proposed method along with energy-based DI can accurately locate the damage, especially in lower speed. Increasing the velocity decreases the accuracy of the proposed method. Therefore, for example, velocity 8 m/s cannot detect the damage location in two scenarios namely; N3D30 and N3N6D40 accurately.

### 5.3. Damage Quantification for Single Damage Scenarios

**Figure 8:**
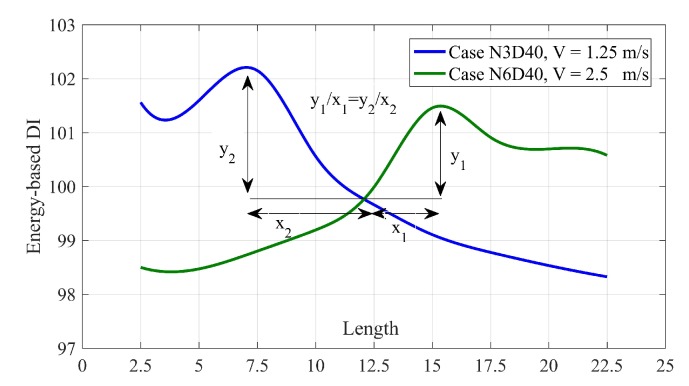
The schematic view of relative slope between the max of spline and the intersection point.

As shown in Figure 6 he spline of DIs for different single damage scenarios intersect each other at a certain point. For the bridge considered in this paper, this intersection is on the length 12 m. As shown in Figure 8, the relative gradient of the spline value at the damage position to the intersection can be used for damage quantification. Table 4 lists the relative slops of splines at the damage position of different single damage scenarios.

As mentioned above, it is clear that the damage cannot be quantified in higher velocities since it cannot be located with high accuracy. Table 4 also shows this inaccuracy. Therefore, at lower velocities, the slope of 0.3 and 0.5 stand for 30% and 40% damage at the bridge.

### 5.4. Considering the Noise

**Figure 9:**
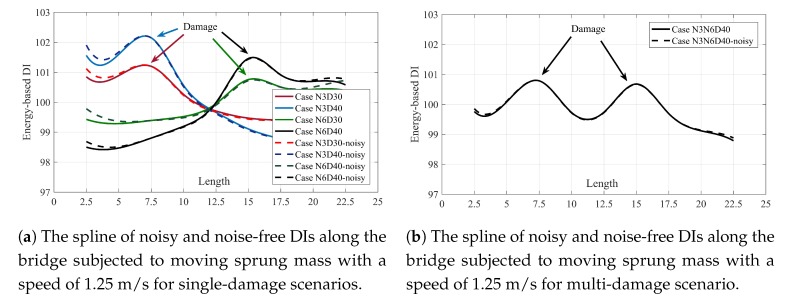
The spline of noisy and noise-free DIs along the bridge subjected to moving sprung mass with a speed of 1.25 m/s. (**a**) single damage scenarios, (**b**) multi-damage scenario.

Due to the lack of access to the experimental result, the proposed method was numerically proved. However, it is proved that temperature variation, road profile and other environmental phenomena may have effect on the results. Therefore, a signal of noise using white noise was randomly produced and manually added to the acceleration signals. To calculate the amount of noise, the root mean square (RMS) ratios of the noise to the signal calculated for all signals. The RMS ratios of the noise to the signal for each node for velocity 1.25 m/s are up to 25%. Increase the speed leads to have more amplitude in acceleration data, so the ratio of noise to the signal will decrease accordingly. The spline of DIs belong to different scenarios and velocities are shown in Figure 9.

As shown in Figure 9, the proposed method is insensitive to the 25% noise at the velocity 1.25 m/s. The effect of this much noise on the results will decrease by increasing the velocity. It is because of decreasing the RMS ratio of the noise in higher velocity. However, the effect of velocity is more than noise, so the proposed method is more sensitive to velocity than noise. Since the SGF is used as de-noising technique, the proposed method still can accurately locate/quantify the damage.

### 5.5. Baseline Estimation

**Figure 10:**
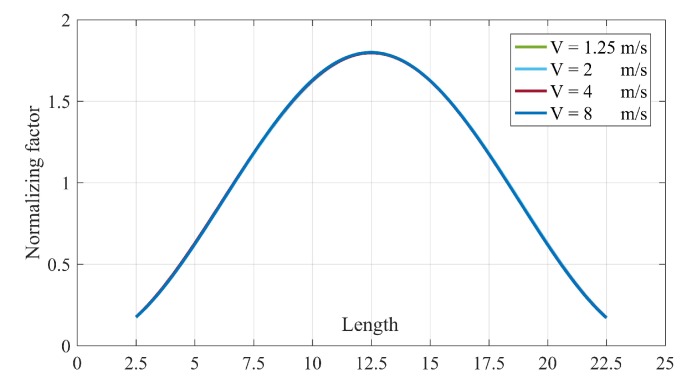
The spline of normalizing factors for different velocities.

As illustrated above, The DIs calculated using Equation (5) successfully locate the damage. In fact, Equation (4) plays the rule of baseline for the proposed damage detection method in Equation (5). Therefore, in this section, a way to estimate Equation (4) for intact bridge is described. Table 5 lists the values of normalization factor for different velocities in noise-free condition. However, based on Figure 9, there is almost no difference between the value of noisy and noise-free conditions. Therefore, only noise-free values are listed in Table 5 and shown in Figure 10.

Figure 10 proves that the spline of different velocities fall on each other. It means that it is possible to use one of them as the baseline for all the velocities between 1.25 to 8 m/s. Since the splines follow a Gaussian distribution, therefore, it is possible to estimate the normalizing factor in other places without accelerometers. These two advantages provide a huge feasibility for the proposed method since keeping a constant velocity before and after damage is very difficult.

## 6. Discussion

This section discusses the effect SGF with different order and span on the results. The changes in natural frequencies is also reported here. All the cases studied here are under moving sprung mass with velocity 1.25 m/s.

### 6.1. The Effect of Different Spans of SGF

**Figure 11:**
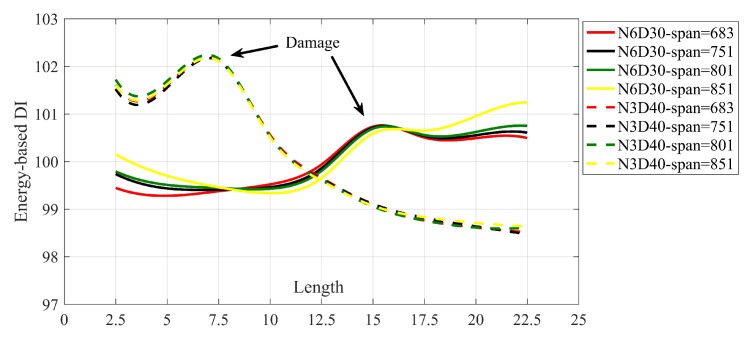
The spline of DIs for different application of SGF with unique span. All the results are for velocity = 1.25 m/s.

To optimize the accuracy of the proposed method, the span of SGF is separately determined for each scenario. This section discusses the effect of selecting a unique span for all cases. Therefore, no matter what is the natural frequency of the bridge, SGF with unique span is applied to all cases. Figure 11 shows the spline of DI calculated using SGF with different spans for cases N3D40 and N6D30.

As shown in Figure 11, all the splines can be used for damage localization. From this figure, it can be seen that there is no difference between different SGF spans in the case N3D40. However, the case N6D30 slowly changes by increasing the SGF span. The peak value in the case N6D30 with the span 683 (red solid line) is easier to recognize than other similar cases. Therefore, for a small percentage of damage, choosing SGF span according to the natural frequency makes results more visible.

### 6.2. The Effect of Different Order of SGF

**Figure 12:**
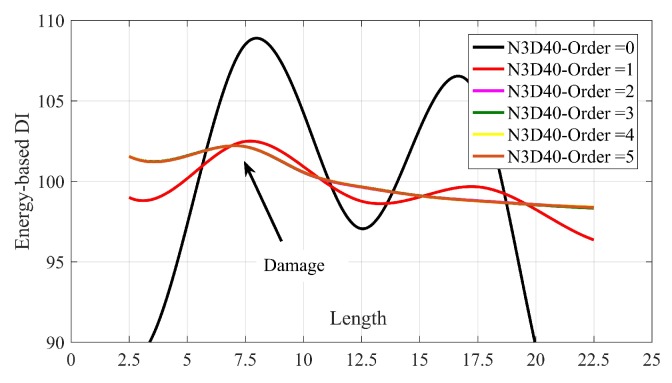
The spline of DIs for using SGF with different orders. All the results are for velocity = 1.25 m/s.

This subsection provides the results of proposed method using SGF with different orders. Figure 12 plotted the spline of DIs for case N3D40 using SGF with order 0 to 5.

Figure 12 illustrates that the proposed method cannot accurately locate the damage using SGF with orders 0 and 1. Although there are maximum in the splines for these two orders at the correct place, with another peak in node 7 (length = 17.5) causes a wrong prediction of existing a multi-damage in the bridge. SGF with order 2 and more falls on each other and gives accurate prediction of damage location. By increasing the order of SGF, the accuracy of damage quantification increases slightly. The use of SGF with order 3 gives enough accuracy to have a precise prediction of damage localization/quantification.

### 6.3. Vehicle-Bridge Interaction

The environmental conditions such as temperature variation can change the natural frequencies. The interaction between vehicle and bridge also causes shift in the natural frequencies of both bridge and vehicle. Moreover, it increases the maximum vertical deflection of the bridge as well. Many factors such as weight, velocity, springs, and damper of the vehicle can increase the vertical deflection of the bridge. There are some literature addressed this effect [43,44,45]. Since this study proposed a damage detection methodology and focused on localizing the damage in the bridge subjected to the moving load, therefore, the effect of vehicle’s parameters (except velocity) are not considered. Only velocity variation was addressed in this paper.

**Figure 13:**
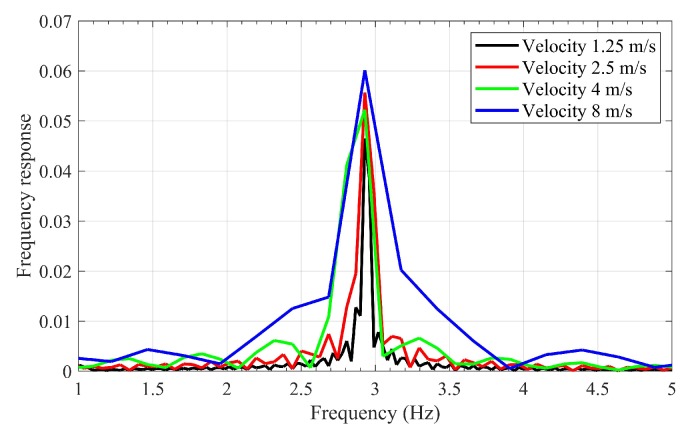
The frequency response of the middle of bridge under moving load with different velocities.

The effect of interaction between bridge and vehicle is illustrated here using two terms namely Dynamic Amplification Factor (DAF) and frequency response of the beam. DAF is defined as below:

(6) $$DAF=\frac{d_{dyn-max}}{d_{sta-max}}$$

in which $d_{dyn-max}$ is the maximum deflection under moving vehicle and $d_{sta-max}=FL^{3}/48EI$ is the maximum static deflection if the vehicle stands in the middle of the bridge. The DAF values are listed in Table 6. This table clearly shows that by increasing the velocity, the maximum amplitude of the acceleration response and the DAF are increased accordingly. Figure 13 shows the frequency response of the beam from acceleration response recorded in the middle of the bridge. Figure 13 also shows the increase of speed leads to have a wider frequency response.

### 6.4. The Effect of Damage on Natural Frequencies

There are various damage detection methods declare that the natural frequency should be changed at least 5% to have an accurate detection of damage. Some of them addressed that even temperature variation sometimes changes the natural frequency more than 5% [1,42]. It is also proved that natural frequency is an aspect of whole of the structure and therefore, a damage (especially a small damage) cannot change the modal parameter such as natural frequencies.

Damage decreases the natural frequencies and makes the bridge softer. Table 7 lists the natural frequencies of the bridge in different cases. Table 7 clearly proves that the proposed method is very sensitive to small changes in the natural frequencies and it might be a good choice for practical applications.

## 7. Conclusions

The main objective of this paper was to locate and quantify the damage in the bridge structure. Therefore, this paper investigated the direct use of SGF to develop a single-stage trend line-based damage detection method for a BHM system. The accuracy of the proposed method was numerically verified using a simply supported beam under moving sprung mass with different velocities. The acceleration data at different places along the beam were recorded, manually polluted with noise, and their trend lines were determined using adjusted SGF. Using normalized energy-based DIs calculated from these trend lines accurately located and quantified the damage. Therefore, it is proved that the trend line of an acceleration signal has signature of damage, so it can be used for damage detection purpose. Additionally, the results prove that the order=3 and span base on the first natural frequency can optimize the accuracy of the proposed method. Therefore, the objective of this study is fully achieved.

The main advantages of the proposed method is as follow:

- Since the SGF is a de-noising technique, the proposed method is essentially insensitive to the noise.
- The proposed method could locate/quantify the damage in noisy/noise-free environment.
- Fitting a Gaussian curve to the normalization factor makes the proposed method as a baseline-free method.
- The proposed method can locate the damage in a multi-damage scenario.

The last but not least, the proposed method does not need neither prior knowledge of damage nor knowledge of input data. Therefore, the proposed method is categorized as output-only damage detection method. This feature makes the proposed method very suitable for practical cases. It should also be mentioned that the main practical limitation for this method is the velocity. Increasing the velocity decreases the accuracy of the proposed method.

## Figures and Tables

**Table 1:** Properties of the simply supported beam.

Properties	Unit	Symbol	Value
**Length**	m	*L*	25
**Mass per unit**	kg/m	*μ*	18,360
**Stiffness**	Nm^2^	*EI*	4.865 × 10^10^

**Table 2:** Properties of the sprung moving mass.

Properties	Unit	Symbol	Value
**Body mass**	kg	$m_{b}$	16,500
**Axle mass**	kg	$m_{t}$	700
**Suspension stiffness**	Nm${}^{-1}$	$K_{s}$	8 × 10^5^
**Suspension damping**	Nm${}^{-1}$	$C_{s}$	2 × 10^4^
**Tire stiffness**	Nm${}^{-1}$	$K_{t}$	3.5 × 10^6^
**Velocity**	m/s	*V*	1.25,2.5,4,8

**Table 3:** Five damage scenarios considered in the numerical model of the simply supported beam.

Scenario	1	2	3	4	5
**Crack depth to the beam height ratio**	30%	40%	30%	40%	40%
**Location**	At node 3	At node 3	At node 6	At node 6	At node 3 and 6
**Name**	N3D30	N3D40	N6D30	N6D40	N3N6D40

Note: The scenarios are designated with N (damage location) and D (ratio). For example, N6D40 refers to a scenario where the damage ratio is 40% at node 6.

**Table 4:** Relative Slope corresponding to different velocities of single damage scenarios.

Scenario	Velocity (m/s)	Slope	Scenario	Velocity (m/s)	Slope
	1.25	0.31	**N3D40**	1.25	0.52
**N3D30**	2.5	0.34		2.5	0.47
	4	0.06		4	0.33
	8	−0.24		8	0.20
	1.25	0.32	**N6D40**	1.25	0.55
**N6D30**	2.5	0.25		2.5	0.46
	4	0.23		4	0.52
	8	0.47		8	0.81
**Average**		0.27	**Average**		0.49

**Table 5:** Normalization factor, $\gamma_{i}$, for all four velocities.

Speed	Node 1	Node 2	Node 3	Node 4	Node 5	Node 6	Node 7	Node 8	Node 9
**1.25**	0.1732	0.6233	1.1773	1.6273	1.8000	1.6286	1.1775	0.6212	0.1718
**2.5**	0.1732	0.6212	1.1759	1.6221	1.7980	1.6287	1.1815	0.6258	0.1736
**4**	0.1761	0.6283	1.1824	1.6270	1.7970	1.6254	1.1746	0.6184	0.1707
**8**	0.1753	0.6228	1.1813	1.6308	1.7997	1.6270	1.1758	0.6175	0.1697

**Table 6:** The max acceleration recorded in the middle of the bridge and the DAF for different velocities.

**Velocity**	1.25 m/s	2.5 m/s	4 m/s	8 m/s
**Max acceleration**	0.0110 m/s${}^{2}$	0.0290 m/s${}^{2}$	0.0324 m/s${}^{2}$	0.0656 m/s${}^{2}$
**DAF**	1.55	1.76	1.96	2.01

**Table 7:** Natural frequencies of the bridge for each case.

Scenarios	1st Natural	Change	2nd Natural	Change	3rd Natural	Change
	Frequency	%	Frequency	%	Frequency	%
**WD**	2.933	—	11.602	—	25.638	—
**N3D30**	2.921	0.4	11.537	0.5	25.622	0.1
**N3D40**	2.909	0.8	11.475	1.1	25.608	0.1
**N6D30**	2.916	0.5	11.577	0.2	25.589	0.2
**N6D40**	2.900	1.1	11.553	0.4	25.533	0.4
**N3N6D40**	2.877	1.9	11.424	1.5	25.502	0.5

## Abbreviations

The following abbreviations are used in this manuscript:

BHM	Bridge Health Monitoring
BSS	Blind Source Separation
DI	Damage Index
RDT	Random Decrement Technique
SGF	Savitzky–Golay filter
SHM	Structural Health Monitoring
SOBI	Second Order Blind Identification
